# Supplementary material for: High cellulose dietary intake relieves asthma inflammation through the intestinal microbiome in a mouse model
Source: PLoS One. 2022 Mar 10;17(3):e0263762. doi: 10.1371/journal.pone.0263762 (PMC8912215; doi:10.1371/journal.pone.0263762)
Supplement: S2 Table — (DOCX) [file pone.0263762.s002.docx]

**S2 Table：Formula of Regular feed**

| **Ingredient g/kg** | **HF (5%)** |
| --- | --- |
| Casein | 200 |
| L-Cystine | 3 |
| Sucrose | 100 |
| Cornstarch | 397 |
| Dyetrose | 132 |
| Soybean Oil | 70 |
| t-Butylhydroquinone | 0.014 |
| Cellulose | 50 |
| Mineral Mix #210025 | 35 |
| Vitamin Mix # 310025 | 10 |
| Choline Bitartrate | 2.5 |
| Calories (kcal/kg) | 2860 |
