# Supplementary material for: High cellulose dietary intake relieves asthma inflammation through the intestinal microbiome in a mouse model
Source: PLoS One. 2022 Mar 10;17(3):e0263762. doi: 10.1371/journal.pone.0263762 (PMC8912215; doi:10.1371/journal.pone.0263762)
Supplement: Attachment — Submitted filename: Response to Reviewers.docx [file pone.0263762.s005.docx]

Dear Editor and Reviewers:

Thank you for your letter. We thank the reviewer and editor for the time and effort that they have put into reviewing the previous version of our manuscript entitled “*High Cellulose dietary intake relieves asthma inflammation through the intestinal microbiome in a mouse model* ”. Your suggestions have enabled us to improve our work. We uploaded the file of the revised manuscript. Accordingly, we have uploaded a copy of the original manuscript with all the changes highlighted by using the track changes mode in MS Word. Appended to this letter is our point-by-point response to the comments raised by the reviewer. The comments are reproduced and our responses are given directly afterward in a different color (red). We would like also to thank you for allowing us to resubmit a revised copy of the manuscript.

Responds to the reviewer`s comments:

Reviewer #4: There are many small problems in the article which need to be carefully revised by the author. Authors should check their manuscripts carefully before submitting them
(1)The author still hasn't corrected the "See Fig Error!".

Response: Thanks for your comment. We apologize for the errors in the manuscript, and we have corrected the formatting errors that have already been mentioned.

(2)The methods are not very detailed, such as how long it takes to build the mouse model and how long the mice will be intervening.

Response: Thanks for your comment. We added a detailed description of the steps to establish the mouse model in the method section.

(3)The picture is very blurry.

Response: Thanks for your comment. We replaced the unclear figures and adjusted them by Preflight Analysis and Conversion Engine (PACE).

Special thanks to you for your good comments.
