# Supplementary material for: High cellulose dietary intake relieves asthma inflammation through the intestinal microbiome in a mouse model
Source: PLoS One. 2022 Mar 10;17(3):e0263762. doi: 10.1371/journal.pone.0263762 (PMC8912215; doi:10.1371/journal.pone.0263762)
Supplement: Attachment — Submitted filename: Response to Reviewers.docx [file pone.0263762.s006.docx]

Dear Editor and Reviewers:

Thank you for your letter.

We were pleased to know that our work was rated as potentially acceptable for publication in Journal, subject to adequate revision. We thank the reviewers for the time and effort that they have put into reviewing the previous version of our manuscript entitled “*High Cellulose dietary intake relieves asthma inflammation through the intestinal microbiome in a mouse model*”.

Based on the instructions provided in your letter, we replaced all the figure files of the manuscript. We sincerely hope that the images uploaded this time will meet the requirements for publication in your magazine.

Responds to the reviewers' comments:

Reviewer #1:

a: Response to comments: Messages such as "(see Fig Error! Reference source not found.)" are scattered throughout the text, please edit.

Response: Thanks for your comment. We apologize for the incorrect writing errors in the manuscript, and we have corrected some of the spelling and formatting errors that have already been mentioned.

b: Response to comments: Were the mice used male, female, both? If only male or female, why was one sex chosen, and how may that affect results?

Response: Thanks for your comment. All the mice used in our experiments are male mice. The reason for this decision is that many studies have pointed out the influence of gender differences on the intestinal microbiome and our focus of research is not to explore the impact of gender differences on the results of the experiment. Therefore, to rule out the possible influence of sex difference, we all selected male mice as experimental subjects.

c: Response to comments : Why was the number of mice chosen used? Was a power analysis or other statistical analysis done to ensure proper numbers of mice?

Response: Thanks for your comment. For the number of mice, we did not do a power analysis to determine it before the experiment. However, we fully considered that only enough samples can reflect the overall level of change, and considering the ability of our laboratory staff, we finally chose the scheme of 10 mice in each group, a total of 30 mice.

Special thanks to you for your good comments.

Reviewer #2:

a: Response to comments: Table 1: I think that the readability of this table could be increased as follow: mean and SD could be put together (mean±SD) and you should add the p-value. Moreover, you can add a legend for this table.

Response: Thank you for your advice on our Table, which makes it easier to read and clearer. We revised the expression of mean and SD and added P-values between the groups in the revised manuscript.

b: Response to comments: Lines 165-167: you could also provide data for differences between N and HF.
Response: Thanks for your suggestion. We added the P-value between groups at the end of the sentence.

c: Response to comments: Line 48: the term “asth ation” is unclear. Please amend it.
Many times (i.e. line 164, 181, 193, 206, etc.), in the paper there is reported the following “Error! Reference source not found”, in correspondence to references to Figures and Tables citations. Please amend it.

Response: Thanks for your comment. We apologize for the spelling and formatting errors in the original version, all of which have been corrected in the new version.

Special thanks to you for your good comments.

Reviewer #3:

a: Response to comments: Line 48, the reviewer is not familiar with asth ation, can the authors clarify what this word is and its meaning. Perhaps it is a typo.

Response: We apologize for the spelling and formatting errors in the original version, all of which have been corrected in the new version.

b: Response to comments: The introduction provides information about some background, but it is jumbled and would benefit from a reorganization and rewrite. Further there does not seem to be an overt purpose or hypothesis established for the study.

Response: Thank you for your comments. We rearrange the paragraphs in introduction, and describe the purpose and results of our experiment.

c: Response to comments: The mice need a more complete description and location of the vendor purchased from. Microbiome/microbiota can vary greatly between vendors and thus influence results of the study.

Response: Thank you for your comments. We clarified the mice strains and the location of the vendor in the revised manuscript. The laboratory animal center of Chongqing Medical university is the experimental animal center of Chongqing National Biological Industry Base and the experimental animal service platform of National Innovative Drug Incubation (Chongqing) Base. By the Chongqing Laboratory Animal Quality Inspection Center and the Chongqing Science and Technology Commission, the center have obtained the production and animal use license of SPF-grade rats and mice, and the animal use license of rabbits, guinea pigs, pigs, dogs, sheep, monkeys, and birds, etc. The quality of experimental animals can be fully guaranteed.

d: Response to comments: Given the perceived purpose is to examine the influence of fiber and asthma, the experimental design seems incomplete, there is no asthma-fiber group. Only normal, asthma and high fiber.

Response: Thank you for your comments. We mentioned the grouping of animals in line 91-92, “the 30 mice were divided into the normal group (N1-N10), asthma group (AS1-AS10), and high-fiber diet group (HF1-HF10).”, and the establishment of models in different groups in line 99-100, “All animals except normal mice were sensitized and challenged with ovalbumin (OVA) to induce airway allergic inflammation.” The HF group was established by high fiber diet intervention in the establishment period of the asthma model.

e: Response to comments: There does not appear to the tables for dietary composition. How were diets altered to account for the high cellulose content? Protein, fat? There was a lot of time spent on the importance of fat in the introduction. This would be critical for understanding interpretation of the results.

Response: Thank you for your suggestion. For the explanation of the feed formula, we put it in lines 96-97, but it lacks the explanation of other important ingredients in feed. Therefore, we decided to upload the complete formula of feed to the journal as a supplement material in the revised manuscript.

f: Response to comments: If all animals were challenged with OVA, then should the groups be renamed?

Response: Thanks for your comment. The grouping and modeling have been explained before. In order to make it easier for readers to understand different groups, we named the normal group N group, the asthma group AS group, and the asthma group with high fiber diet intervention, and we named it HF group based on the main intervention measures.

g: Response to comments: The authors do not appear to have a scoring outline in place to understand how the IHC data was analyzed to note differences in staining.

Response: Thanks for your comment. In order to determine the infiltration of inflammatory cells in the lungs of mice, we used H&E staining instead of IHC, and then observed the morphology and structure of bronchioles and the changes of infiltration of inflammatory cells around them under light microscope.

h: Response to comments: The authors have 16S rDNA in the V4-V5 regions, this should be rRNA. While one must extract the DNA it then must be prepared for rRNA analysis.

Response: Thanks for your comment. We corrected the inaccurate expression in the method section

i: Response to comments: SCFA in animal models are analyzed using cecal contents so that fecal pellets can be used for microbiota.

Response: Thanks for your comment. We know that SCFAs are mainly absorbed in the cecum and enter the blood circulation. The content of cecum can reflect the changes of SCFAs in mice more directly. In our experiment, considering that SCFAs may be a potential biomarker in human body, and the most convenient way to detect SCFAs in human body is to detect the content of SCFAs in fresh feces. So in order to simulate this situation, we collected fresh feces of mice in the experiment, and immediately put them into the refrigerator at - 80 ℃ for storage, to minimize the possible volatilization of SCFAs.

j: Response to comments: The statistical analysis is very vague and does not employ appropriate techniques to analyze m data of this fashion.

Response: Thanks for your comment. We apologize for the vague expression in the data analysis section. In the revised manuscript, we redescribed different data analysis methods for different experimental contents.

Special thanks to you for your good comments.
